# Clinical and Genetic Study of X-Linked Juvenile Retinoschisis in the Czech Population

**DOI:** 10.3390/genes12111816

**Published:** 2021-11-18

**Authors:** Bohdan Kousal, Lucia Hlavata, Hana Vlaskova, Lenka Dvorakova, Michaela Brichova, Zora Dubska, Hana Langrova, Andrea L. Vincent, Lubica Dudakova, Petra Liskova

**Affiliations:** 1 Department of Paediatrics and Inherited Metabolic Disorders, First Faculty of Medicine, Charles University and General University Hospital in Prague, 128 08 Prague, Czech Republic; ocnigenetika@vfn.cz (B.K.); hlavata.lucia@gmail.com (L.H.); hana.vlaskova@lf1.cuni.cz (H.V.); lenka.dvorakova@lf1.cuni.cz (L.D.); lubica.dudakova@lf1.cuni.cz (L.D.); 2 Department of Ophthalmology, First Faculty of Medicine, Charles University and General University Hospital in Prague, 128 08 Prague, Czech Republic; michaela.brichova@vfn.cz (M.B.); zora.dubska@vfn.cz (Z.D.); 3 Department of Ophthalmology, Faculty of Medicine in Hradec Kralove, Charles University and University Hospital Hradec Kralove, 500 05 Hradec Kralove, Czech Republic; hana.langrova@fnhk.cz; 4 Department of Ophthalmology, New Zealand National Eye Centre, University of Auckland, Auckland 1142, New Zealand; a.vincent@auckland.ac.nz

**Keywords:** *RS1*, X-linked retinoschisis, novel variant, uveitis, steroid treatment

## Abstract

The aim of this study was to identify *RS1* pathogenic variants in Czech patients with X-linked retinoschisis (XLRS) and to describe the associated phenotypes, including natural history, in some cases. Twenty-one affected males from 17 families were included. The coding region of *RS1* was directly sequenced and segregation of the identified mutations was performed in available family members. In total, 12 disease-causing variants within *RS1* were identified; of these c.20del, c.275G>A, c.[375_379del; 386A>T], c.539C>A and c.575_576insT were novel, all predicted to be null alleles. The c.539C>A mutation occurred de novo. Three patients (aged 8, 11 and 19 years) were misdiagnosed as having intermediate uveitis and treated with systemic steroids. Repeat spectral domain optical coherence tomography examinations in four eyes documented the transition from cystoid macular lesions to macular atrophy in the fourth decade of life. Four individuals were treated with topical dorzolamide and in two of them, complete resolution of the cystic macular lesions bilaterally was achieved, while one patient was noncompliant. Rebound phenomenon after discontinuation of dorzolamide for 7 days was documented in one case. Misdiagnosis of XLRS for uveitis is not uncommon; therefore, identification of disease-causing variants is of considerable benefit to the affected individuals.

## 1. Introduction

X-linked retinoschisis (XLRS; OMIM number 312700) is a rare vitreoretinal dystrophy with an estimated prevalence ranging from 1 in 5000 to 20,000 affected inhabitants [1].

Clinically, the disease is characterized in males by early-onset visual loss and bilateral foveal schisis due to splitting of the inner retinal layers; 33–60% of patients with XLRS also have peripheral retinoschisis [2,3,4]. Complications, such as retinal detachment, occur in 5–22% cases, and intraretinal haemorrhage within a schisis cavity or vitreous haemorrhage is present in approximately one third of patients [5,6,7]. Less commonly observed clinical findings include diffuse white retinal flecks [8], Coats disease–like exudative retinopathy, perivascular sheathing, peripheral dendriform lesions and vitreous veils [7,9].

Unless secondary complications occur, XLRS typically shows slow bilateral progression resulting in macular atrophy by the fifth or sixth decade of life [10]. Marked differences, even in members of individual families, can be found [11]. Heterozygous female carriers are typically unaffected [2]. Patients with XLRS may show a benefit from systemic or topical treatment with carbonic anhydrase inhibitors (CAIs) for cystic macular lesions and mid-peripheral retinoschisis [12,13,14].

XLRS is caused by mutations in *RS1* encoding retinoschisin [15,16], which plays an important role in maintaining the retinal structure as a cell adhesion protein between photoreceptors and bipolar cells [17,18]. To date, more than 250 pathogenic variants in *RS1* have been reported [19,20].

In this study, we have identified disease-causing variants in 17 Czech families with XLRS. We also performed deep phenotyping in our cohort, including natural history, response to topical dorzolamide treatment and optical coherence tomography angiography (OCTA). The study documents the spectrum of genetic and phenotypic variability in XLRS patients resulting in misdiagnosis in three cases for a previously unreported population.

## 2. Materials and Methods

Affected and unaffected members from 17 Czech families of European descent with XLRS participated in the study. The study was approved by the Ethics Committee of General University Hospital in Prague (reference no. 34/19) and adhered to the tenets set out in the Helsinki Declaration. All participants or their legal guardians signed informed consent prior to the inclusion into the study. Ophthalmic evaluation comprised measurements of best-corrected visual acuity (BCVA) and intraocular pressure, perimetry, fundus photography (Clarus 700 and FF 450 plus IR, Carl Zeiss Meditec AG, Jena, Germany), spectral domain optical coherence tomography (SD-OCT; Spectralis, Heidelberg Engineering GmbH, Heidelberg, Germany and Spectral OCT/SLO, OTI Ophthalmic Technologies Inc., Toronto, Canada), OCTA (Spectralis OCT2, Heidelberg Engineering GmbH), and full-field electroretinography (ERG; RetiPort + mf ERG system, Roland Consult, Brandenburg, Germany) using DTL-electrodes, according to the standards of International Society for Clinical Electrophysiology of Vision [21]. All amplitudes and implicit times were compared with the mean values ± SD of normal age-matched controls.

Genomic DNA was isolated from peripheral blood samples using standard methods. All six exons of *RS1* and intron–exon boundaries were bidirectionally Sanger sequenced. Primer sequences used were as previously reported [22] using NM_000330.3 as the reference sequence. The presence of the identified variants in first-degree relatives was tested also by conventional sequencing. The Genome Aggregation Database v2.1.1 (gnomAD; http://gnomad.broadinstitute.org/, accessed on 17 October 2021) was searched to determine variant frequency [23]. Because of the rarity of XLRS, only variants with minor allele frequency ≤0.005 were further evaluated for potential pathogenicity and cross-referenced with published literature, public archive of interpretations of clinically relevant variants ClinVar (https://www.ncbi.nlm.nih.gov/clinvar/, accessed on 9 November 2021) and the *RS1* LOVD database (https://databases.lovd.nl/shared/genes/RS1, accessed on 17 October 2021). The pathogenicity of the detected variants was evaluated according to the American College of Medical Genetics and Genomics and the Association for Molecular Pathology (ACMG-AMP) recommendations [24].

## 3. Results

Twenty-one Czech males from 17 not knowingly related families (Supplementary Figure S1) clinically diagnosed with XLRS were included in the study.

Molecular diagnosis was achieved in all families; 12 different *RS1* pathogenic variants were identified, of which 5 were novel (Table 1). All novel mutations were predicted to be null alleles, either generating a stop codon or a frameshift with subsequent introduction of a premature or delayed stop codon (Table 1, Supplementary Figure S1). Novel *RS1* variants were unique to the families, except for c.20del, which was observed recurrently in families F1 and F2, who interestingly originated in nearby geographical regions (less than 30 km from each other) suggesting a common founder.

Seven mutations, 6 missense and 1 frameshifting, have been previously reported associated with XLRS in multiple families (Table 1). Only one variant c.544C>T had an entry in the gnomAD v2.1.1 population dataset (1 allele out of 182,642—observed in a heterozygous female).

Segregation analysis within the families showed that c.539C>A; p.(Ser180*) occurred de novo in the proband from family F10 (Supplementary Figure S1). All variants were classified as pathogenic according to the ACMG-AMP guidelines as they have been either previously reported to be associated with XLRS and/or lead to a loss of function, and were absent in gnomAD v2.1.1 male samples.

Clinical ophthalmic data of the affected males are summarized in Supplementary Table S1. Sixteen subjects were evaluated at repeated intervals with a mean follow-up of 9.8 years (range 0.6–34 years).

**Figure 1:**
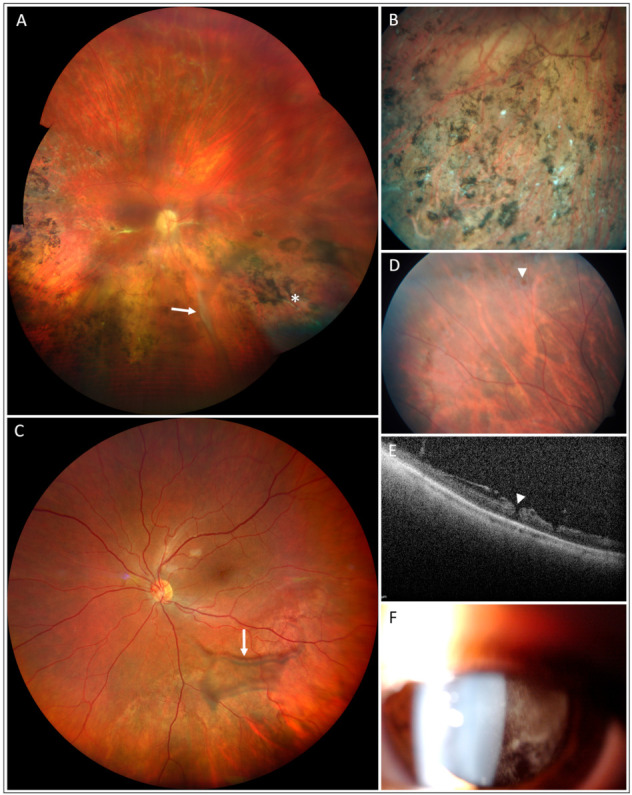
Less frequent clinical findings in patients with XLRS. Vitreous veil (arrow), vascular sheathing (asterisk) and pigmentary changes in the right eye of individual F1-III:1 aged 39 years (**A**,**B**). Vitreous veil of the left eye (arrow) in individual F17-II:1 aged 35 years (**C**). Retinal hole in the right eye (arrowhead) of the individual F5-II:1 aged 11 years (**D**,**E**). Vitreous haemorrhage in the left eye of the individual F13-II:1 aged 9 years (**F**).

**Figure 2:**
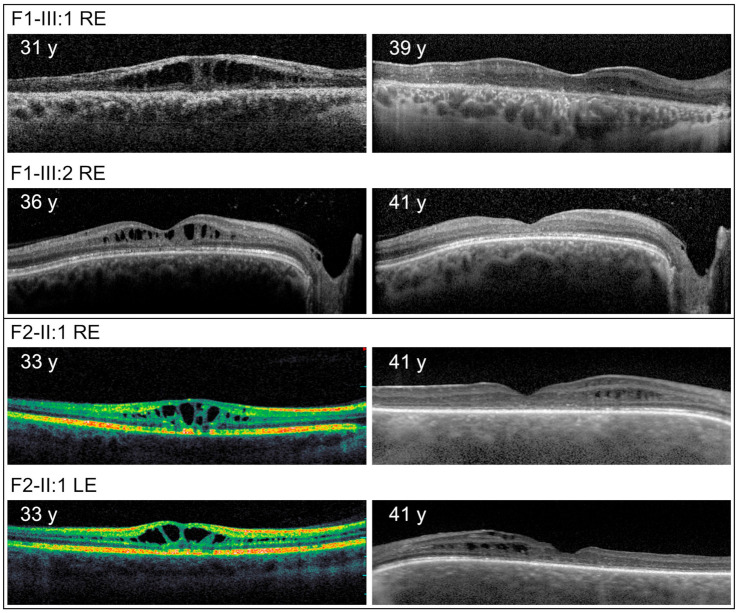
Repeated imaging with optical coherence tomography in individuals with XLRS documenting natural history disease course from cystic changes to macular atrophy. y = years, LE = left eye, RE = right eye.

At the most recent examinations, typical macular retinoschisis was detected in 15 individuals (29 eyes), atrophic changes in the macula in 6 individuals (11 eyes), and peripheral retinoschisis in 7 (13 eyes). Additional rarer phenotypes included vitreous veils in 3 (4 eyes), sheathing of retinal vessels, retinal holes in 2 patients (3 eyes) and vitreous haemorrhage observed in 1 patient (2 eyes) (Figure 1). Progression of macular retinoschisis to macular atrophy was documented using SD-OCT in 4 eyes of 4 individuals; none had been treated with topical dorzolamide (Figure 2).

Notably, findings in four patients revealed atypical clinical phenotypes, which led to diagnostic difficulties. Individual III:1 from family F1 presented at the age of 30 years with subcapsular cataract, dense vitreous opacities, migration and clumping of retinal pigment epithelium (RPE) in the macula and the periphery, perivascular sheathing and white spicules in the right eye (Figure 1A,B), while in the left eye only macular retinoschisis was documented. Fluorescein angiography however excluded vasculitis. Complete ophthalmic imaging of the proband is shown in Supplementary Figure S2. A diagnosis of XLRS was subsequently made using ERG, which demonstrated a typical selective reduction of the b-wave of the standard scotopic response, with preservation of the a-wave resulting in a reduction of the b/a quotient, and subsequently confirmed by genetic testing. Both the proband, and his male cousin with a typical XLRS phenotype (Figure 2, Supplementary Table S1), were found to possess the novel variant c.20del in *RS1*.

Investigation of Individual II:1 from family F5 was undertaken after a bilateral BCVA decrease was noted at a routine paediatric check-up at the age of 10 years. Due to the presence of cystoid macular edema with vascular sheathing, a diagnosis of intermediate uveitis was made, and systemic steroids were introduced when he was aged 11 years. Full thickness retinal holes and retinoschisis in the fundus periphery were also documented in both eyes (Figure 1D,E, Supplementary Figure S3). A range of tests were performed to identify the underlying cause of the observed ocular pathology, including MRI and laboratory examination of cerebrospinal fluid. The patient was treated with systemic methylprednisolone for 3.5 years until the patient was 14 years old, at which point ERG was performed, demonstrating a negative b-wave suggesting a diagnosis of XLRS. Subsequent molecular genetic testing identified hemizygosity for a known c.187T>C mutation in *RS1*. After obtaining a molecular diagnosis, steroid treatment was gradually tapered down and discontinued.

Individual II:1 from family F10, aged 19 years, was treated elsewhere with systemic methylprednisolone for 4 months because of bilateral cystoid macular edema, until referred to our tertiary care setting where an XLRS diagnosis was made, subsequently confirmed by the identification of a novel pathogenic variant c.539C>A in *RS1*. Based on the positive genetic test, steroid administration was gradually decreased and stopped.

Individual II:1 from family F13 was treated elsewhere for cystoid macular oedema and intermediate uveitis with systemic steroids from 9 years of age. He also received a dexamethasone intravitreal implant bilaterally. When examined by the authors at the age of 10 years, a typical star-shaped macular lesion was found in the right eye and resolving vitreous haemorrhage in the left eye (Figure 1F). No signs of inflammation were present. SD-OCT showed bilateral typical schitic cavities (Supplementary Figure S4). Molecular genetic analysis identified the novel *RS1* variant c.575_576insT predicted to be pathogenic.

Available clinical documentation in individuals F4-II:1 and F14-III:3 permitted comparison of BCVA over more than three decades. No significant decrease was noted.

**Figure 3:**
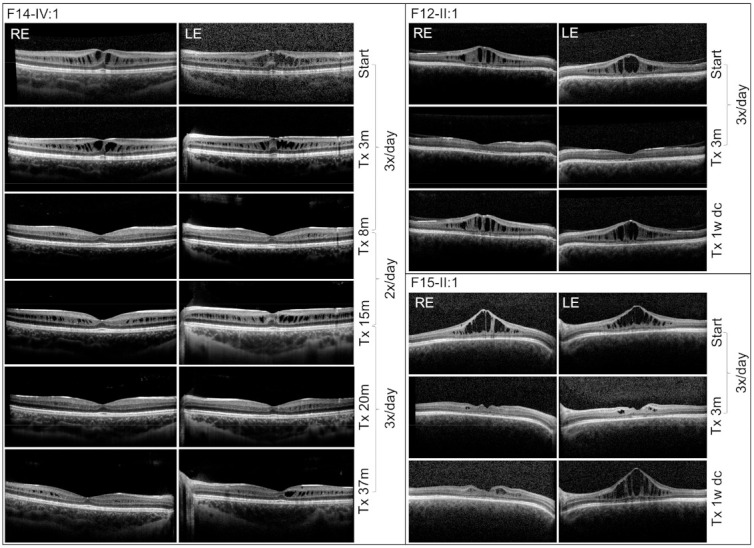
Effects of dorzolamide treatment in three XLRS patients as documented by SD-OCT. Results of individual treatment dosing as well as the length of the therapy are shown. Note rebound phenomena in patient F15-II:1 only after one week of discontinuation of drop administration. dc = discontinued, m = months, Tx = treatment, w = week.

Therapeutic intervention for the schitic macular lesions with CAIs (dorzolamide, 2% drops three times a day) was started in four individuals (F8-II:1, F12-II:1, F14-IV:1, F15-II:1). Decrease of retinal thickness was observed on SD-OCT in all eyes. Patient F14-IV:1, aged 7 years at the start of the treatment, had the longest follow-up (37 months) during which we were able to document complete resolution of the schitic cavities; however, after changing the treatment regimen to twice a day, rebound occurred. After returning to dosing three times a day improvement was observed (Figure 3). Individuals F12-II:1 and F15-II:1 responded well, with none or very few cystic changes noted 3 months after the start of the treatment; however, discontinuation of the treatment for only 7 days led to a marked rebound phenomena (Figure 3). Two patients (F8-II:1, F14-IV:1) noticed subjective improvement of visual functions, which was also documented on BCVA measurements (Supplementary Table S1).

Patient F16-II:1 underwent OCTA in addition to other examinations. An irregular foveal avascular zone and flow loss within the deep capillary plexus corresponding to the distribution of the schisis were noted (Supplementary Figure S5).

We also comprehensively examined one heterozygous carrier (F14-III:2). No fundus pathology was noted both on wide-field imaging nor SD-OCT.

## 4. Discussion

In this study, we report the clinical and molecular genetic findings in 17 families with XLRS ascertained in the Czech Republic. In addition to the identification of novel pathogenic variants, our study provides further data on XLRS responsivity to topical dorzolamide treatment and the natural history of the disease.

Five novel disease-causing mutations in *RS1* were identified, all predicted to be loss of function alleles. Four pathogenic variants, c.20del, c.33_36del, c.305G>A and c.544C>T, were observed in more than one family, although haplotyping to either confirm or disprove a founder effect was not undertaken. Given the close geographical origin, we hypothesise that at least families F1 and F2 are likely to share a common ancestor.

In family F10, the c.539C>A variant in *RS1* was present only in the proband and not in the genomic DNA of his mother, indicating a likely de novo origin, albeit mosaicism cannot be entirely excluded. Interestingly, the rate of de novo *RS1* mutations is low, and to the best of our knowledge, previously only reported in seven cases with XLRS [16,25,29,30,35].

Three probands were misdiagnosed with uveitis and treated with steroids, administered systemically in two patients, and bilateral dexamethasone intravitreal implants were inserted in one individual.

The progressive nature of XLRS has been debated [10,36,37,38]. Available clinical documentation suggested that the disease is relatively stable with no major deterioration unless proliferative vitreoretinopathy develops.

In this study, we have also documented on SD-OCT, in five eyes of three individuals, the transition from cystic changes to macular atrophy, interestingly without any major effect on BCVA. OCTA imaging, which is a relatively new method, was similar to previously described cases and documented perifoveal microvascular changes. The usefulness of this method for monitoring disease progression or treatment response is yet to be established [39,40,41,42].

To conclude, the diagnosis of XLRS can be challenging due to phenotypic variability. XLRS may be misdiagnosed as uveitis, and in these cases in particular, the combined modalities of electrophysiology and molecular genetic testing can be crucial in confirming a diagnosis. Longitudinal records of BCVA in XLRS seem to be stable, even in older individuals who are documented to develop atrophic changes in the macula.

## Figures and Tables

**Table 1:** *RS1* disease-causing mutations identified in Czech families with X-linked retinoschisis.

Family	DNA Level	Protein Level	ClinVar Interpretation/VCV Accession	References
F1, F2	c.20del	p.(Gly7Alafs*119)	Not present	Novel
F3, F4, F15	c.33_36del	p.(Leu11Phefs*114)	Pathogenic/VCV000098944.7	[25]
F5	c.187T>C	p.(Cys63Arg)	Not present	[26]
F6	c.275G>A	p.(Trp92*)	Not present	Novel
F7, F8	c.305G>A	p.(Arg102Gln)	Pathogenic/VCV000009896.11	[11,25,27,28,29]
F9	c.375_379del	p.(Asp126Glufs*16)	Not present	Novel
F10	c.539C>A	p.(Ser180*)	Not present	Novel
F16	c.421C>T	p.(Arg141Cys)	Pathogenic/VCV000098959.9	[11,25,30]
F11, F17	c.544C>T	p.(Arg182Cys)	Pathogenic/VCV000098986.3	[25,29,31,32]
F12	c.574C>T	p.(Pro192Ser)	Pathogenic/VCV000098990.4	[15,25,32]
F13	c.575_576insT	p.(Ile194Hisfs*70)	Not present	Novel ^#^
F14	c.637C>T	p.(Arg213Trp)	Pathogenic/VCV000099009.5	[25,29,33,34]

NM_000330.3 was used as the reference sequence; ^#^ Novel at DNA level; at protein level p.(Ile194Hisfs*70) has been reported.

## Data Availability

Not applicable.

## Supplementary Materials

The following are available online at https://www.mdpi.com/article/10.3390/genes12111816/s1, Figure S1: Pedigrees of 17 Czech families with X-linked retinoschisis and segregation of the identified RS1 mutations, Figure S2: Clinical findings in individual F1-III:1 aged 39 years, Figure S3: Clinical findings in individual F5-II:1 aged 15 years, Figure S4: Clinical findings in individual F13-II:1 aged 9 years, Figure S5: Clinical findings in individual F16-II:1 aged 40 years, Table S1: Summary of clinical data in 21 male individuals with X linked retinoschisis.
