# Supplementary material for: Clinical and Genetic Study of X-Linked Juvenile Retinoschisis in the Czech Population
Source: Genes (Basel). 2021 Nov 18;12(11):1816. doi: 10.3390/genes12111816 (PMC8623540; doi:10.3390/genes12111816)
Supplement: Supplementary file 1 [file genes-12-01816-s001.zip › genes-1450719-supplementary.pdf]

Supplementary Materials

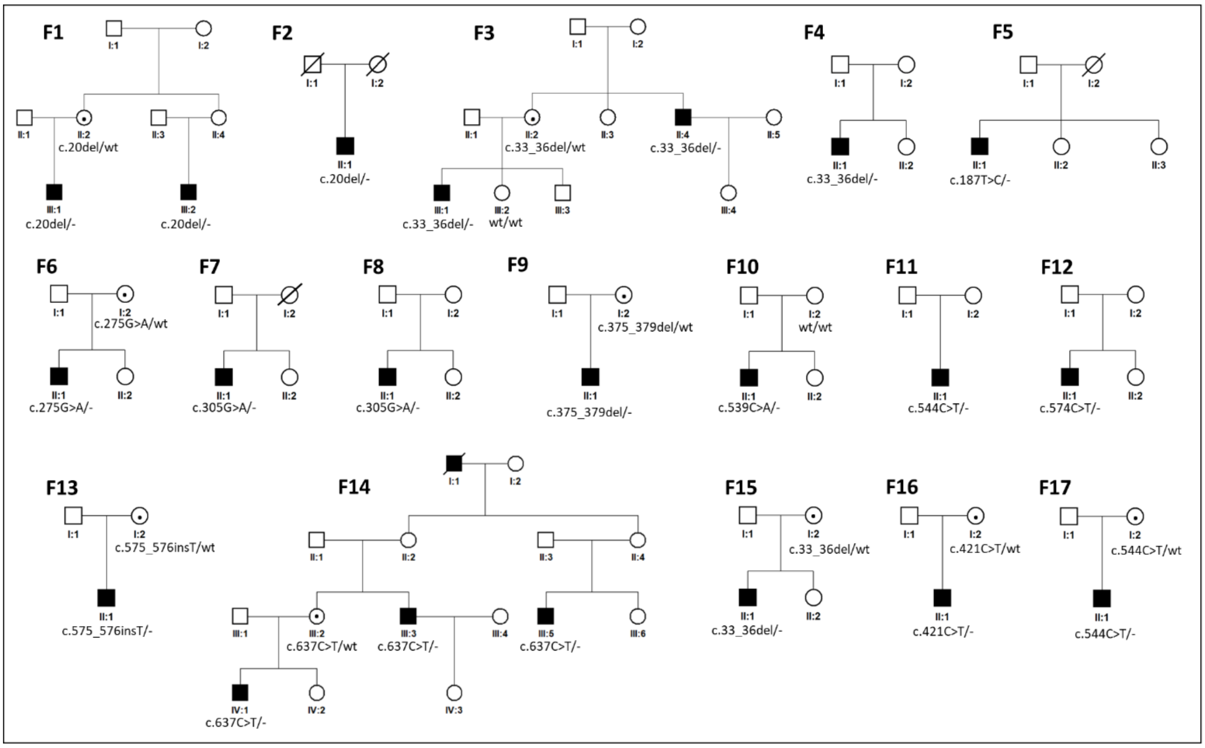

Supplementary Figure S1. Pedigrees of 17 Czech families with X-linked retinoschisis and segregation of the identified *RS1* mutations. Affected males are represented by black squares. Only genetically tested females and obligate carriers are shown as a circle with a dot. In family 10 the mother of the proband had two wild-type (wt/wt) alleles, hence a *de novo* origin of the mutation in her son is likely.

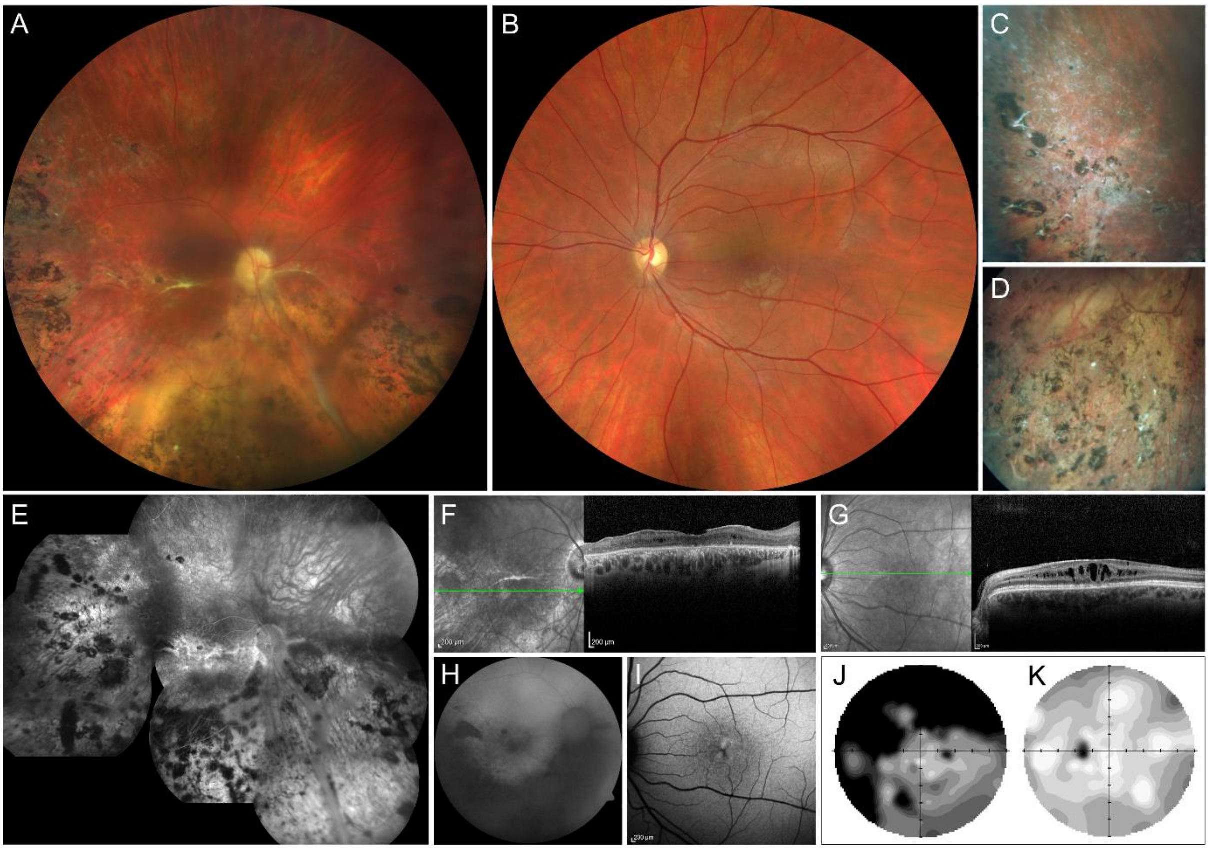

Supplementary Figure S2. Clinical findings in individual F1-III:1 aged 39 years. Ultra-widefield fundus photograph of the right (**A**), and left (**B**) eye, temporal periphery in detail right (**C**), and left (**D**) eye. Fluorescein angiography in the right eye (**E**). SD-OCT of the right (**F**), and left (**G**) macula. Fundus autofluorescence in the right eye (**H**, **I**). Static perimetry in the right (**J**), and left (**K**) eye.

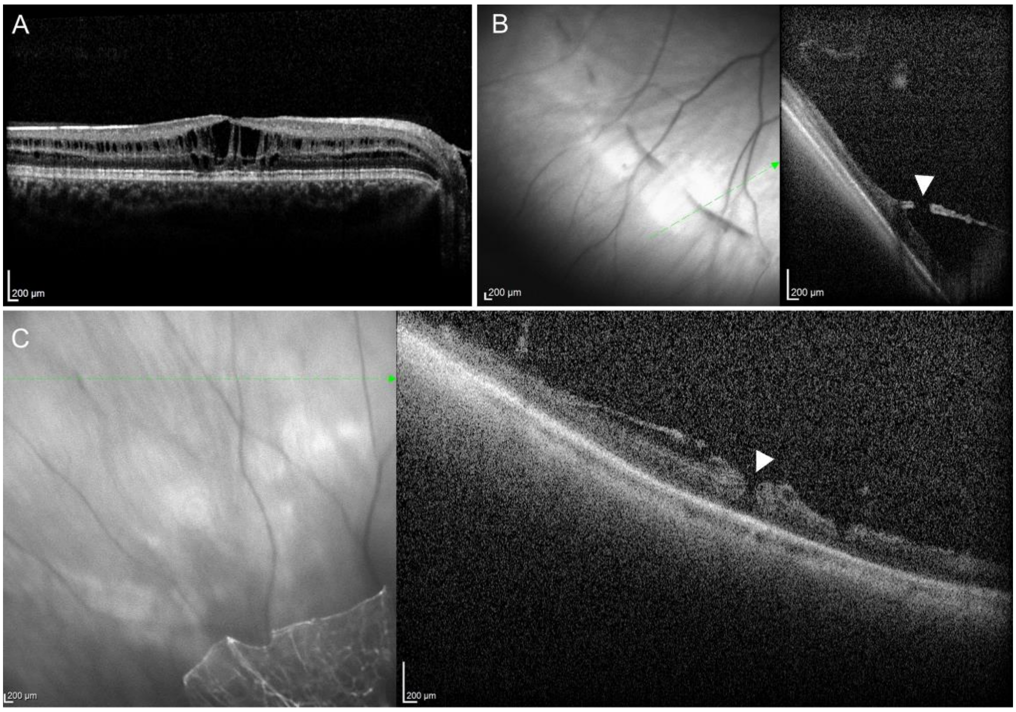

Supplementary Figure S3. Clinical findings in individual F5-II:1 aged 15 years. SD-OCT of the right macula (**A**), inner leaf hole (arrowhead) seen in inferotemporal retinal quadrant of the right eye (**B**), full thickness hole (arrowhead) found in superotemporal retinal quadrant of the right eye (**C**).

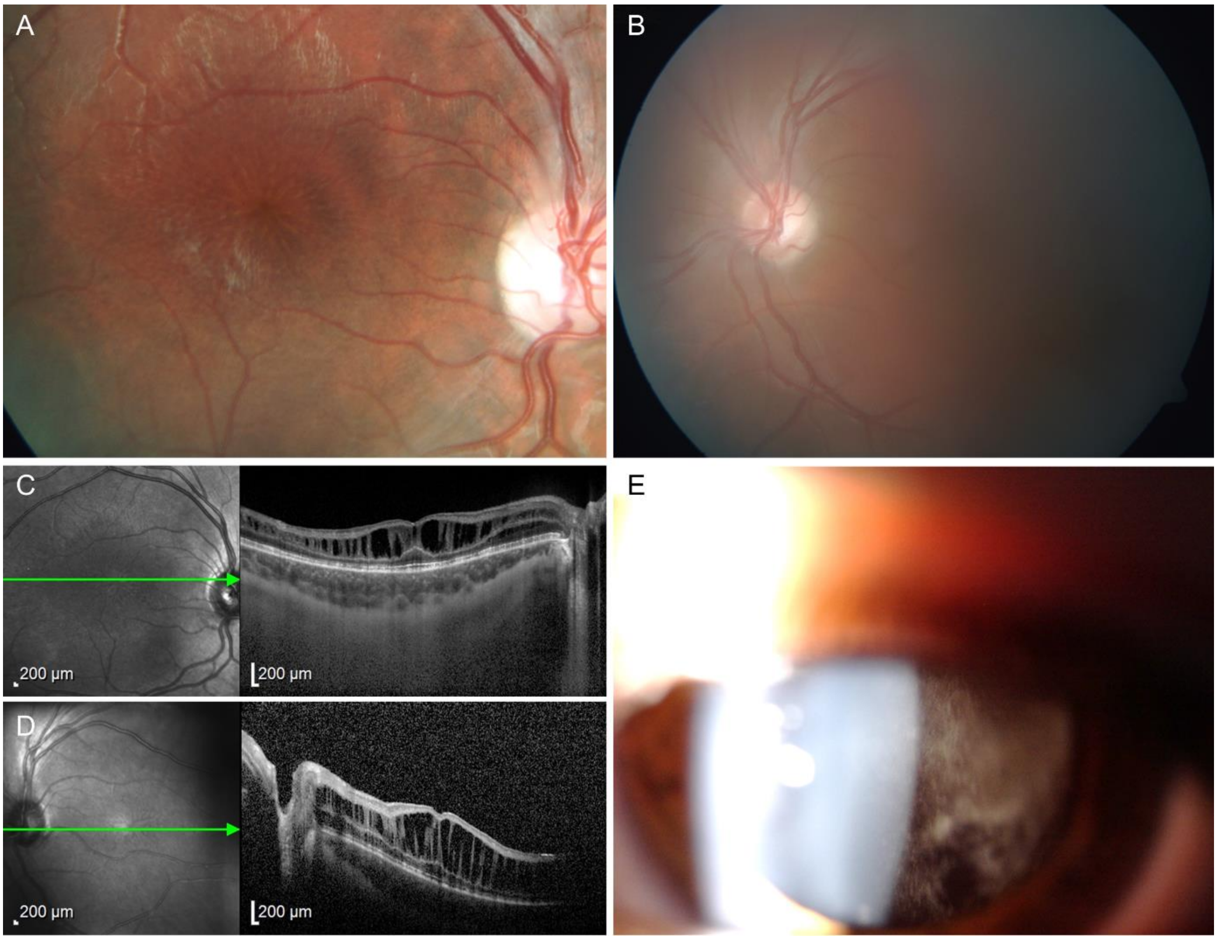

Supplementary Figure S4. Clinical findings in individual F13-II:1 aged 9 years. Fundus photographs showing the right macula (**A**), and blurred details of the left eye due to vitreous haemorrhage (**B**). SD-OCT of the right (**C**), and left (**D**) macula. Vitreous haemorrhage of the left eye as observed by slit lamp examination (**E**).

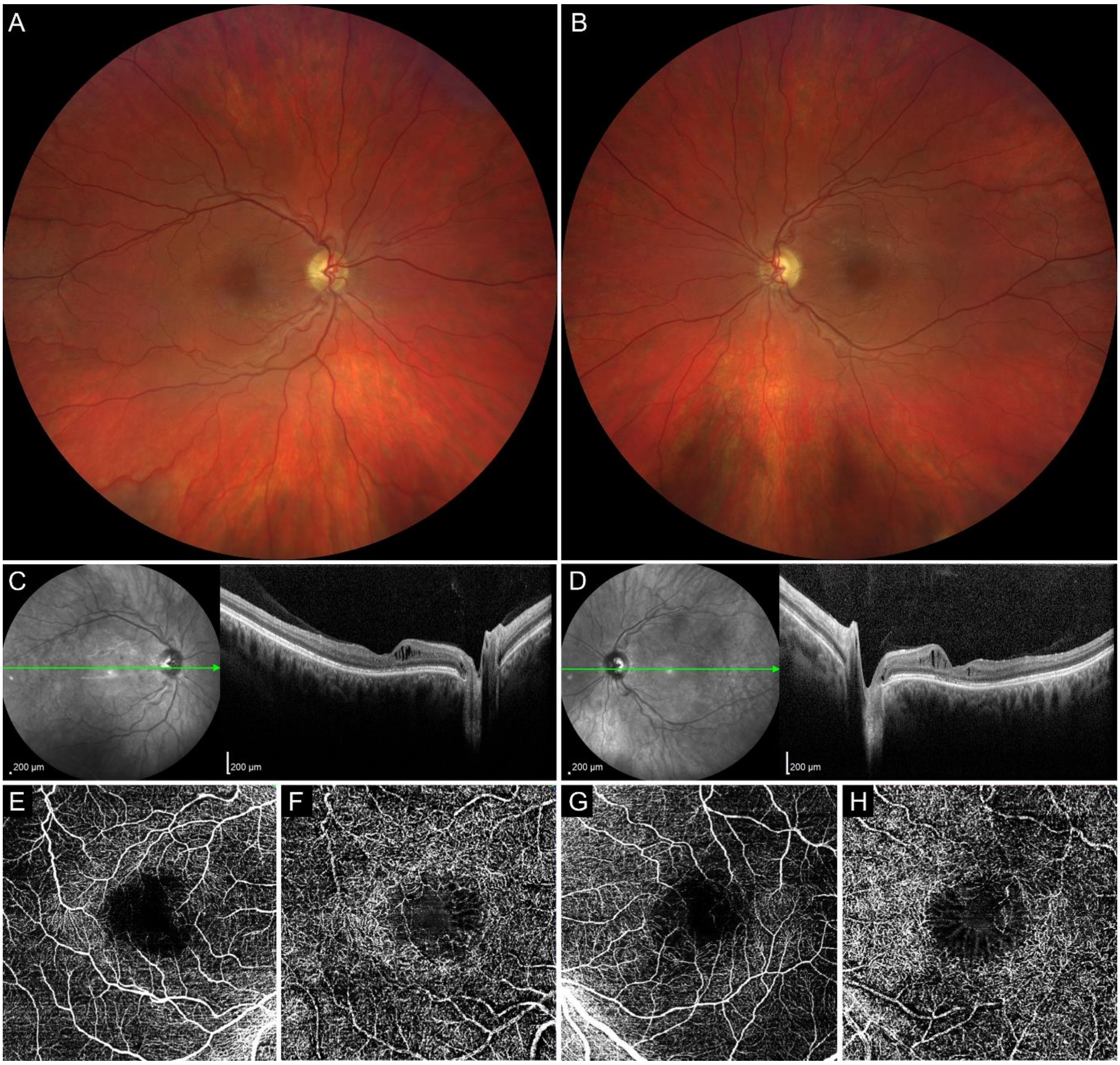

Supplementary Figure S5. Clinical findings in individual F16-II:1 aged 40 years. Ultra-widefield fundus photograph of the right (**A**), and left (**B**) eye. SD-OCT of the right (**C**), and left (**D**) macula. OCTA imaging of the superficial (**E**), and deep (**F**) vascular complex of the right eye and superficial (**G**), and deep (**H**) vascular complex of the left eye, note irregular foveal avascular zone and flow loss within the deep capillary plexus corresponding to the distribution of the schisis.

Supplementary Table S1. Summary of clinical data in 21 male individuals with X linked retinoschisis

Family/ Individual	Follow -up	Age (y)	BCVA		Schitic spaces in the macula on SD-OCT/layer		Other findings	
			RE	LE	RE	LE	RE	LE
F1-III:1	9 y	30	0.15	0.5	Y••/INL	Y••/INL+GCL	Vitreous veils, irregular pigmentation, vascular sheathing	Nil
		39	0.1	0.5	N (atrophy)	Y••/INL+GCL	Vitreous veils, irregular pigmentation, vascular sheathing	Nil
F1-III:2	5 y	36	0.4	0.05	Y•/INL	Y••/INL	Nil	Nil
		41	0.32	0.05	N (atrophy)	N (atrophy)	Nil	Nil
F2-II:1	14 y	26	0.5	0.5	Y••/INL+GCL	Y••/INL	Peripheral retinoschisis	Peripheral retinoschisis, inner retinal holes, laser photocoagulation scars
		40	0.4	0.5	Y••/INL+GCL	Y••/INL	Peripheral retinoschisis	Peripheral retinoschisis, inner retinal holes, laser photocoagulation scars
F3-II:4	-	46	0.1	0.1	N (atrophy)	N (atrophy)	Peripheral retinoschisis, macular irregular pigmentation	Peripheral retinoschisis, macular irregular pigmentation
F3-III:2	3 y	12	0.2	0.3	Y/UA	Y/UA	Nil	Nil
		15	0.16	0.25	Y•••/INL+GCL	Y•••/INL+GCL	Nil	Nil
F4-II:1	33 y	9	0.4	0.4	UA	UA	UA	UA
		42	0.32	0.25	N (atrophy)	N (atrophy)	Macular irregular pigmentation	Macular irregular pigmentation
F5-II:1	6 y	11	0.66	0.4	Y••/INL+ONL	Y••/INL+ONL	Peripheral retinoschisis, inner and full thickness retinal holes, vascular sheathing, retinal haemorrhage	Peripheral retinoschisis, inner and full thickness retinal holes, vascular sheathing, retinal haemorrhage
		17	0.66	0.33	Y•••/INL+ONL	Y•••/INL+ONL	Peripheral retinoschisis, inner and full thickness retinal holes, vascular sheathing	Peripheral retinoschisis, inner and full thickness retinal holes, vascular sheathing
F6-II:1	3 y	17	0.4	0.4	Y•••/INL	Y•••/INL	Vitreous veils, peripheral retinoschisis	Vitreous veils, peripheral retinoschisis
		20	0.4	0.4	Y•••/INL	Y•••/INL	Vitreous veils, peripheral retinoschisis	Vitreous veils, peripheral retinoschisis
F7-II:1	15 y	33	0.4	HM	UA	UA	Diffuse myopic chorioretinal atrophy, peripheral retinoschisis, white spiculations	Diffuse myopic chorioretinal atrophy, peripheral retinoschisis, RPE migration
		48	0.1	LP	UA	UA	Diffuse myopic chorioretinal atrophy, proliferative vitreoretinopathy, peripheral retinoschisis, white spiculations	Diffuse myopic chorioretinal atrophy, proliferative vitreoretinopathy, chorioretinal scar

F8-II:1	2.5 y	48	0.2	0.2	Y•••/INL+ONL+GCL	Y•••/INL+ONL+GCL	Nil	Nil
		50	0.25	0.4	Y•••/INL+ONL+GCL	Y•/INL	Nil	Nil
		51	0.3 (Tx)	0.4 (Tx)	Y•/INL	Y•/INL	Nil	Nil
F9-II:1	14	32	0.25	0.25	Y/UA	Y/UA	Peripheral irregular pigmentation	Peripheral irregular pigmentation
		41	0.1	0.1	Y•INL	Y•/INL+GCL	Peripheral irregular pigmentation	Peripheral irregular pigmentation, macular white dots
		46	0.05	0.05	Y•/INL+GCL	Y•/INL+GCL	Peripheral irregular pigmentation	Peripheral irregular pigmentation, macular white dots
F10-II:1	-	19	0.32	0.32	Y•••/INL+ONL+GCL	Y•••/INL+ONL+GCL	Nil	Nil
F11-II:1	-	30	0.3	0.63	Y•••/ONL+INL	Y•••/ONL+INL	Peripheral retinoschisis	Peripheral retinoschisis
F12-II:1	10 m	23	0.2	0.16	Y•••/INL	Y•••/INL	Peripheral metallic sheen	Peripheral metallic sheen
		24	0.2 (Tx)	0.16 (Tx)	Y•••/INL	Y•••/INL	Peripheral metallic sheen	Peripheral metallic sheen
F13-II:1	7 m	8	0.2	0.2	Y•••/INL+ONL	Y•••/INL+ONL	Vitreous opacities	Vitreous opacities
		9	0.5	0.6	Y•••/INL+ONL	Y•••/INL+ONL	Decolorized vitreous haemorrhage	Vitreous haemorrhage
F14-III:3	34 y	19	0.25	0.5	UA	UA	Nil	Nil
		53	0.3	0.4	N (atrophy)	N (atrophy)	Nil	Peripheral choroidal nevus
F14-III:6	-	39	0.3	0.5	N (atrophy)	N (atrophy)	Nil	Macular white dots
F14-IV:1	4 y	6	0.8	0.7	Y••/INL	Y••/INL	Macular white dots	Nil
		10	0.9 (Tx)	0.9 (Tx)	Y•/INL	Y•/INL+GCL	Macular white dots	Nil
F15-II:1	7 m	21	0.25	0.4	Y•••/INL+GCL	Y•••/INL	Nil	Nil
		21	0.25 (Tx)	0.32 (Tx)	Y•/INL	Y•••/INL	Nil	Nil
F16-II:1	12 y	28	0.2	0,32	UA	UA	Nil	Nil
		40	0.3	0.4	Y•/INL	Y•/INL	Nil	Nil
F17-II:1	-	35	0.16	0.63	Y•••/INL+ONL	Y••/ONL+INL	Peripheral retinoschisis	Peripheral retinoschisis, vitreous veil

GCL = ganglion cell layer, HM = hand movement, INL = inner nuclear layer, LE = left eye, m = months, ONL = outer nuclear layer, RE = right eye, SD-OCT = spectral domain optical coherence tomography, Tx = treatment with dorzolamide, UA = unavailable data, y = years, Y = yes
